# The Efficacy of Hemostatic Radiotherapy for Advanced Malignancies Assessed by World Health Organization Bleeding Status

**DOI:** 10.7759/cureus.19939

**Published:** 2021-11-27

**Authors:** Atsuto Katano, Hideomi Yamashita

**Affiliations:** 1 Radiology, The University of Tokyo Hospital, Tokyo, JPN

**Keywords:** hemostatic, bleeding, retrospective analysis, radiation therapy, palliative care

## Abstract

Purpose

This retrospective study aimed to evaluate the efficacy of radiotherapy for the palliation of bleeding symptoms of advanced malignancies.

Patients and methods

We included 36 consecutive patients treated for hemostatic intent by radiotherapy at our institution, from August 2013 to January 2019. Patient bleeding status was assessed according to the World Health Organization (WHO) bleeding status before and after radiotherapy.

Results

We identified 36 consecutive patients, consisting of 14 men and 22 women. The median follow-up period after radiotherapy for all cases was 2.5 months (range: 0.4-37.7 months), and the one-year OS was 47.1% (95% confidence interval: 23.5%-67.6%). Of the 36 patients, 29 (81%) showed improvement by one point or more in the WHO bleeding status. A total of 15 patients received 30 Gy in 10 fractions with a biologically effective dose (BED10) of 39 Gy (high BED arm). The remaining 21 patients had received radiotherapy with a BED10 of less than 39 (low BED arm, range of BED10: 11.2-30 Gy). In the high BED arm, 14 of the 15 patients (91%) showed improvement on the WHO bleeding status. In the low BED arm, 15 of the 21 patients (71%) showed improvement on the WHO bleeding status. There was no statistically significant difference in the improvement rate between the high and low BED arms (p = 0.200).

Conclusion

There is no universal standard to evaluate the hemostatic effect of palliative radiotherapy. In this retrospective study, we investigated the clinical outcomes of hemostatic irradiation as assessed by WHO bleeding status.

## Introduction

Radiation therapy is an important tool for palliative treatment of pain and other symptoms associated with cancer invasion and metastasis. Major indications of palliative radiotherapy include pain relief, control of bleeding, fungating lesion and ulceration, dyspnea, obstruction of visceral cavities, and reduction of problematic tumors due to space occupation [1-2]. Radiation therapy for painful bone metastases is a typical example of palliative radiation therapy and is the area of palliative radiation therapy for which the most evidence is available [3]. According to ENABLE studies, appropriate palliative care has been linked to significant improvements in quality of life and prolonged survival [4-5].

Approximately 10% of patients with advanced-stage cancer have bleeding symptoms [6]. Symptoms vary according to the bleeding site but include epistaxis, bloody sputum, hematemesis, hematuria, bloody stools, and vaginal hemorrhage. Constant bleeding has a significant impact on a patient's quality of life [7]. Additionally, clinically serious bleeding requires burdensome medical interventions, such as blood transfusions, which significantly impact the length of hospital stay and medical costs [8].

Although the hemostatic effect of palliative radiotherapy is well-known, there is no universal consensus as to how to assess the effect. Then, this retrospective study aimed to evaluate the efficacy of radiotherapy for palliation of bleeding symptoms by a quantitative score. Furthermore, we analyzed the potential relationships between patient factors and overall survival, as well as the prescription dose and bleeding control of hemostatic radiotherapy.

## Materials and methods

In this retrospective study, we included consecutive patients treated with radiotherapy for hemostatic intent at our institution between August 2013 and January 2019. Informed consent was obtained by all the patients. This study was approved by the Research Ethics Committee of the Faculty of Medicine of the University of Tokyo (Approval number: 3372-5). Patient bleeding status was assessed using the World Health Organization (WHO) bleeding status before and after radiotherapy [9]. All patients meeting the following inclusion criteria; (i) patients suffered from clinically significant malignant bleeding (WHO bleeding status of 2 or higher), (ii) referred to our department for palliative radiotherapy, and (iii) first time of palliative radiotherapy. Patients with insufficient medical records, such as lost to follow-up, were excluded from the study.

All patients underwent palliative radiotherapy with photon linear accelerators using three-dimensional conformal radiotherapy. Planning computed tomography (CT) image data for radiation therapy was reconstructed in 5 mm slice thickness. The CT data were sent to a treatment planning system (Pinnacle^TM^, Philips, Amsterdam, Netherlands). The clinical target volume (CTV) included the bleeding tumor site. The planning target volume included the CTV with added clinically sufficient margins. The radiation oncologist determined the dose and fractionation of radiotherapy at the time of treatment. The biologically equivalent dose (BED10) was used to evaluate the equivalent radiation doses in other radiotherapy treatment schedules. BED10 was calculated by nd (1+d/10), where n is the number of fractionations and d is the dose per fraction.

The R statistical package (R Foundation for Statistical Computing, Vienna, Austria) was used for all statistical analyses. Overall survival (OS) was calculated using the Kaplan-Meier method. Univariate Cox proportional hazards analysis was performed for the risk analysis of overall survival. Fisher's exact analysis to test the null hypothesis was used to compare the two categorical variables. Statistical significance was set at p < 0.05.

## Results

We identified 36 consecutive patients, consisting of 14 men and 22 women between 32 and 91 years of age (median age: 71 years). The patient characteristics for this study were summarized in Table 1.

**Table 1:** Clinical characteristics Background characteristics of 36 consecutive patients in this study

Characteristics		N	percentage
Age		Median: 71 (32-91)	
Sex	Male	14	(39)
	Female	22	(61)
Karnofsky Performance Status	90%	1	(3)
	80%	5	(14)
	70%	10	(28)
	60%	11	(31)
	50%	9	(25)
Pathology	Adenocarcinoma	19	(53)
	Squamous cell carcinoma	8	(22)
	Others	9	(25)
Primary or metastasis	Primary	22	(61)
	Metastasis	14	(39)
Previous radiotherapy	No	27	(75)
	Yes	9	(25)
Location	Genitourinary	18	(50)
	Gastrointestinal	7	(19)
	Skin or lymph nodes	8	(22)
	Others	3	(8)

The doses and fractionations used in this study are listed in Table 2. Fifteen patients received 30 Gy in 10 fractions with a BED10 of 39 Gy (high BED arm). The remaining 21 patients had received radiotherapy with a BED10 of less than 39 (low BED arm). A baseline KPS score of 70 or less was observed in 83% of the patients.

**Table 2:** The details of radiotherapy The doses and fractionation of palliative radiotherapy used in this study. BED10: biologically equivalent dose, which was calculated by nd (1+d/10), where n is the number of the fractionation and d is the dose per fraction.

Radiotherapy schedules	BED10 [Gy]	N	percentage
30Gy/10fr	39	15	(42)
20Gy/5fr	28	10	(28)
8Gy/1fr	14.4	3	(8)
8Gy/2fr	11.2	3	(8)
16Gy/2fr (one week apart)	28.8	2	(6)
20Gy/4fr	30	1	(3)
15Gy/3fr	22.5	1	(3)
21Gy/7fr	27.3	1	(3)

**Figure 1:**
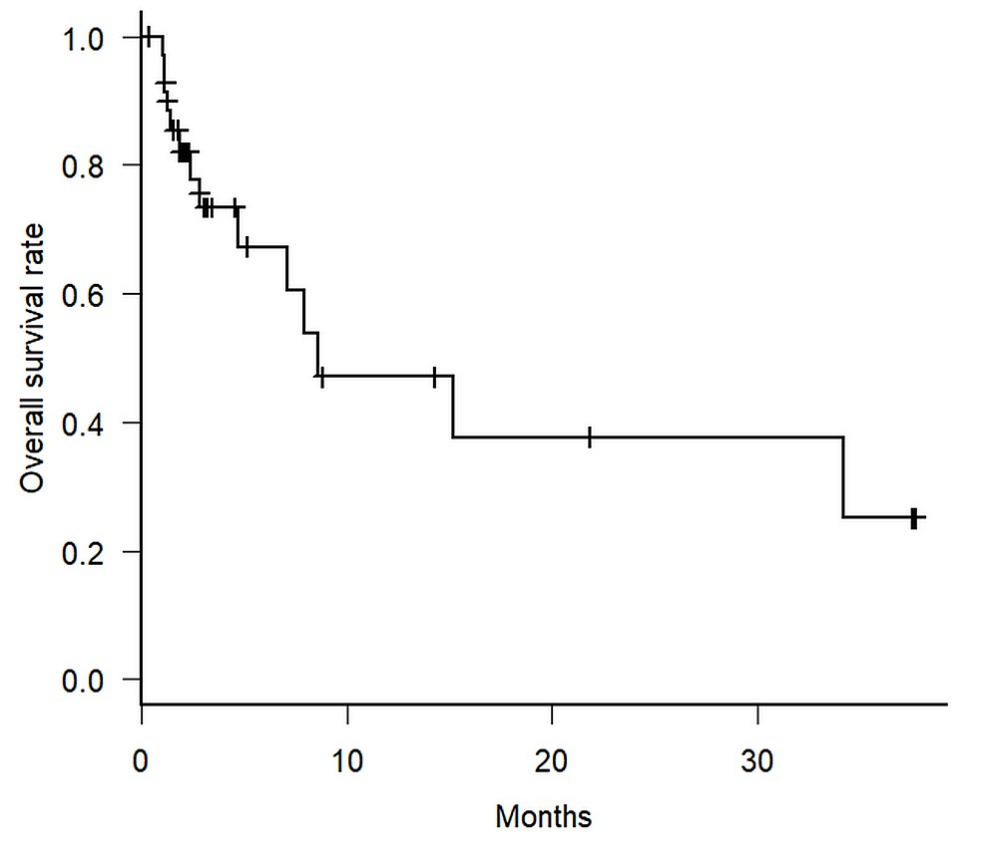
Kaplan-Meier curve for overall survival Kaplan-Meier curve for overall survival in patients of this study. Vertical bars indicate censored cases.

The pathological diagnosis of the patient was adenocarcinoma in 19 patients, squamous cell carcinoma in eight patients, and others in nine patients. Nine patients had a previous radiotherapy history of the target lesion. The median follow-up period for all cases was 2.5 months (range: 0.4-37.7 months), and the 1-year OS was 47.1% (95% CI: 23.5%-67.6%). Figure 1 shows the Kaplan-Meier curve of overall survival for all patients in this study. Univariate Cox hazard analysis revealed no significant differences in OS between patient characteristics, as shown in Table 3.

**Table 3:** Prognosis factor for overall survival Univariate Cox proportional hazard analysis of overall survival; WHO: World Health Organization

Parameter	Hazard Ratio (95% CI)	P-value
Age: < 70 vs. others	0.82 (0.29-2.37)	0.7196
Sex: male vs female	1.11 (0.38-3.24)	0.8457
KPS: > 60 vs others	2.29 (0.76-6.91)	0.1428
pathology: Adenocarcinoma vs others	0.77 (0.27-2.21)	0.6257
primary or meta: Primary vs metastasis	1.54 (0.53-4.49)	0.4297
Previous Radiotherapy: No vs Yes	1.66 (0.52-5.27)	0.3932
Location: Genitourinary vs others	1.79 (0.61-5.24)	0.2864
WHO bleeding status: >2 vs others	0.81 (0.17-3.75)	0.7855
BED: >30 vs others	1.71 (0.58-5.07)	0.3301

Information regarding pre and post-radiation WHO bleeding status is presented in Table 4.

**Table 4:** WHO bleeding status Comparison of WHO bleeding status scores pre- and post-radiotherapy; WHO: World Health Organization

	pre-radiotherapy		post-radiotherapy	
WHO bleeding status	N	percentage	N	percentage
0	0	(0)	10	(28)
1	0	(0)	14	(39)
2	18	(50)	7	(19)
3	16	(44)	5	(14)
4	2	(6)	0	(0)

Out of the 36 patients, 29 (81%) showed improvement by one point or more in the WHO status (1 point, 10 patients; 2 points, 18 patients; 3 points, 1 patient). In the high BED arm, 14 of the 15 patients (91%) showed improvement on the WHO status. In the low BED arm, 15 of the 21 patients (71%) showed improvement on the WHO status. There was no statistically significant difference in this rate between the high and low BED arms (p = 0.200).

## Discussion

The results of the present palliative hemostatic radiotherapy were consistent with those reported in the past. Sapienza analyzed 112 patients who received radiotherapy for symptomatic relief of hemorrhagic tumors and reported that treatment response was observed in 89% of the patients in a single-center retrospective study [10]. Cihoric analyzed 62 patients (including one patient with benign disease) who received radiation therapy to relieve bleeding tumor symptoms. A retrospective analysis of these patients (including one patient with benign disease) showed that bleeding status improved in 54 (87%) of patients treated with radiotherapy, and 39 (63%) of these patients had a complete response to bleeding [11]. Rasool reported that palliative hemostatic radiotherapy was administered to 25 patients with various types of cancer, and 22 patients (88%) responded [12].

There is controversy as to whether there is a correlation between the hemostatic response and radiation therapy dose. Viani conducted a meta-analysis on radiotherapy for bleeding response in patients with advanced-stage gastric cancer [13]. According to their analysis, a significant improvement in the hemostatic rate was identified in the higher BED treatment group. On the other hand, Chaw et al. revealed that short-course radiotherapy, a type of low BED radiotherapy, is an effective treatment that can provide symptom relief from gastric cancer [14]. Kawabata also reported the effectiveness and safety of short-or low-dose radiotherapy for bleeding gastric cancer [15]. In the present study, there was no statistically significant difference between the high and low BED arms, but the high BED arm tended to have a better hemostasis rate than the low BED arm, which was compatible with a previous report [16].

For better clinical outcomes, concurrent chemotherapy with palliative radiotherapy has been used in several studies. Asakura et al. reported that 22 of 30 patients (73%) responded to radiotherapy for advanced-stage gastric cancer bleeding. They reported that 12 patients received concurrent chemoradiotherapy and had a significantly lower rebleeding rate than those who received RT alone (p = 0.001) [17]. Although the response rate of radiation therapy with chemotherapy might be high, it is more invasive than monotherapy and should be limited to patients with good performance status. Patients in the terminal stage of the disease are more likely to choose radiation alone because of their lower performance status and general condition.

Interventions other than radiotherapy also have a favorable effect on malignant bleeding. Song et al. reported successful endoscopic hemostasis in 83% of 106 advanced gastric cancer patients with bleeding [18]. Transcatheter arterial embolization is a treatment option for bleeding in advanced-stage cancer [19]. The use of Mohs ointment is also effective for local bleeding from surface malignancies [20]. Surgical removal of the bleeding site is the most direct method but requires careful patient selection because of the high perioperative risk [21]. It is important to select the appropriate treatment modality for each patient based on each patient's general condition, prognosis, surgical tolerance, complications, and bleeding site.

There are several limitations to our study. First, the most significant limitation of this study was the limited number of cases, which limited the amount of statistical processing. Second, the retrospective analysis of the data obscured the radiotherapy decision process regarding dose and fractionation at the initiation of treatment. Third, our data relied on medical records, which may have been insufficient to accurately describe the characteristics and clinical events of all patients.

## Conclusions

There are no universal criteria for evaluating the hemostatic effect of palliative radiotherapy. In order to optimize the management of hemostatic radiotherapy, it is important to establish a reliable and universal assessment index. This retrospective study investigated the clinical outcomes of hemostatic irradiation assessed by WHO bleeding status. We considered that the WHO bleeding status might be one of the useful assessment scales of hemostatic radiotherapy. Further accumulation of evidence is needed to reveal the adaptation and validation of this scale.
